# Being active 1½ years after hip fracture: a qualitative interview study of aged adults’ experiences of meaningfulness

**DOI:** 10.1186/s12877-020-01666-w

**Published:** 2020-07-29

**Authors:** Birgit Rasmussen, Claus Vinther Nielsen, Lisbeth Uhrenfeldt

**Affiliations:** 1 Department of Physio and Occupational Therapy, Horsens Regional Hospital, Sundvej 30, 8700 Horsens, Denmark; 2 Department of Public Health, Aarhus University, Bartholinsallé 2, 8000 Aarhus C, Denmark; 3 DEFACTUM, Aarhus, Central Denmark Region Denmark; 4 Regional Hospital West Jutland, Herning, Denmark; 5 Faculty of Nursing and Health Science, Nord University, Universitetsalléen 11, 8049 Bodø, Norway

**Keywords:** Hip fracture, Aged, Physical activity, Qualitative, Rehabilitation, Well-being, Hermeneutics

## Abstract

**Background:**

Being active is vital and a source of well-being. However, 18 months after hip fracture (HF), progress seems to have come to a halt. Aged adults may feel vulnerable, experiencing ongoing dependency and limited possibilities for socializing. How they experience the meaningfulness of being active during these circumstances is unknown. The aim of this study was to explore experiences of the meaningfulness of being active for aged adults 18 months after HF.

**Methods:**

A phenomenological-hermeneutic methodology based on the philosophies of Heidegger and Gadamer was applied. Data were collected using individual interviews conducted in participants’ homes. The study was part of a longitudinal study, and three former interviews helped build trusting relationships with participants and focus the semi-structured interview guide. An existential theory of well-being and suffering considering health to be a balancing of mobility and dwelling was applied.

Participants were nine aged adults 65 years or older with pre-fracture dependency included in the study 18 months earlier while still in hospital after HF. The interpretation was a process of analyzing data by moving between the parts and the whole as a means of gaining a deeper understanding and continuously testing pre-understandings. The analysis followed five steps: a) getting a sense of the whole b) delineating and condensing meaning units, c) interpreting meaning units, d) relating to study purpose, and e) developing themes and sub-themes.

**Results:**

Two main themes emerged. The main-theme “Feeling the continuity of life “had four sub-themes: “Gratitude for present possibilities, ““Connected with earlier life-experience, ““Thoughtfully managing vulnerability, “and “Belonging with other people. “The main-theme “Feeling vulnerable “had two sub-themes: “Thwarted “and “Sad and regretting lost continuity in life.“.

**Conclusions:**

Eighteen months after HF, aged adults seem to be struggling on their own to be active in meaningful ways. To maintain hope, relieve the strain in everyday life, and maintain a sense of safety and self-confidence, they may need help. However, to avoid suffering, there is a need to balance additional training and a struggle for progress with well-being experiences in terms of feeling gratitude, restoring a sense of normality, and feeling kinship with other people.

## Background

Applying a human perspective [1], this study is concerned with aged adults’ individual experiences of the meaningfulness of being active 18 months after hip fracture (HF). Based on an existential world-view, meaningfulness can be an experience of being active as part of everyday life [2], and coming to understand how well-being is possible in challenging life situations [1]. The challenge of experiencing meaningfulness after HF is the lost capability to manage everyday life tasks [3] and the diminished possibilities for socializing [4]. For 40–60% of aged adults, the challenge is never to regain prior mobility; for around 30%, increased dependency in self-care; and for more than 50%, lost ability to independently go to places out of walking distance [5]. These challenges are especially prevalent for aged adults with pre-fracture limitations of their mobility [6]. For aged adults in general, a sense of meaningfulness seems to be related to integrating valued activities and being active as a natural part of everyday life: e.g. being in familiar places [7], socializing, or being outdoor [8].

Although recovery after HF takes place primarily during the first year, some progress is still possible beyond (Alarcon 2011), and it seems possible to increase the numbers of social activities aged adults take part in [9]. However, many feel challenged by a permanent loss of physical functions [10, 11] and a feeling of vulnerability after HF; it may be a struggle to be active and on the edge of what is possible to endure. Owing to persistent pain, tiredness, or fear of falling, aged adults can lose self-confidence [12], and they can experience a feeling of being imprisoned because of restricted social life and inactivity [13]. Striving to find meaning and be active after HF, relationships with other people are essential to avoid losing courage, and well-being may be connected with being able to be content with limited possibilities for being active [14].

When aged adults are inactive after HF [15], this adds to the risk of diminished well-being and can lead to further dependency, loss of functioning, and premature death [16–18]. After completing a rehabilitation program, there may be a need for additional and better qualified rehabilitation including training and knowledge on how to improve own physical functions [19]. Growing evidence shows that prolonged rehabilitation in municipal programs, including modification of the home, intensified exercises, education and social support, can increase possibilities for being active and increase a sense of well-being after HF. However, consensus and guidelines for municipality-based rehabilitation do not exist [20–23].

### The authors’ pre-understanding

The authors´ ontological pre-understanding is Heidegger’s philosophy and concerns the meaningfulness of being active in-the-world, evolving into a unified experience of past experiences carried into the present, while being concerned about the unknown future [2]. Facing bereavement and loss, the meaning of being active changes, and the awareness of life conditions including the finitude of life becomes present [2]. As human beings, we are open toward possibilities in life and feeling responsible for our own being-in-the-world. When our being is challenged by the vicissitudes of life conditions, and when personal longings and beliefs are confronted, we can develop the skill to make independent decisions to improve our lives [24]. Based on Heidegger’s ontology, Todres and Galvin described meaningfulness to be an intertwined experience of suffering and well-being [1]. Well-being at its deepest core is an experience of dwelling and mobility in everyday life situations. Dwelling is to allow things to be the way they are in the present, and mobility is a drive toward new possibilities and a better future [1]. In contrast, experiences of being stuck and feeling homeless in the present are related to suffering. Temporality, spatiality, embodiment, inter-subjectivity, identity and mood are interwoven nuances of life-world experiences; although present at the same time some are in the foreground, while others are the background, giving coherence in life [25].

The authors’ epistemological pre-understanding is evidence on aged adults’ experiences after HF, clarifying how positive experiences of feeling able to pursue wanted activities may coexist with negative aspects of living with limitations of physical ability and restrictions in everyday life [13]. Amid experiences of illness, vulnerability, and disability, reconciliation with the fact that life after HF is not the same as before can be an essential resource and restore a sense of meaningfulness. Existential experiences of belonging, feeling at peace, or maintaining courage seem to be essential to experience that being active is meaningful [12].

The professional pre-understanding of the first author (BR) is rooted in 3 decades of physiotherapeutic practice experience working with in-hospital rehabilitation of aged adults; the experience from conducting three former interview-rounds with participants of this study, adding depth to the discussion section; and the significance of person-oriented care as central to healthcare practice [26]. Contextual pre-conditions in Denmark are the fast-track in-hospital program after HF followed by a written rehabilitation plan effectuated in municipalities after discharge from hospital [27]. Municipality-based interventions in Denmark include a reablement perspective [28], and preventive home-visits on regular basis in the homes of aged adults are implemented [29]. Access to rehabilitation, homecare, assistive devices and day-care centers is partly or completely cost-free for the individual.

Limitation of physical functions due the HF may still exist 18 months after HF, and healthcare providers do experience that it is a challenge for aged adults to be active in meaningful ways. Knowledge is scarce on long-term experiences of being active after HF at a time when progress no longer seems possible. To prevent permanent or increasing limitation in well-being and functioning after HF, it is vital to clarify the challenges and the possibilities existing in aged adults’ everyday life 18 months after HF. This study will develop ontological knowledge that can be used in healthcare providers’ practice when planning and creating early and ongoing interventions in hospitals and in the municipalities to support aged adults in being active in meaningful ways after HF.

### Aim

The aim of this study was to explore and understand aged adults’ everyday life experiences of the meaningfulness of being active 18 months after a HF.

## Methods

The study is phenomenological-hermeneutic, a research tradition emphasizing the meaning people attach to their experiences and actions [2]. Based on existential philosophy, interpretation is an inevitable part of the research process and we as researchers cannot escape our history and look at things objectively [30]. Meaning appears within the researchers’ horizons when a new understanding arises from a fusion between what is already known about the phenomena and what is added from the lived experiences revealed by interacting with the participants [31]. To gain a deeper understanding beyond the clinical perception of being active, a thorough challenge of one’s own pre-understandings is applied using the hermeneutic rule of moving cyclically between parts and wholes throughout the research process, gradually reaching a deeper understanding that includes the horizon of the participants [31].

**Fig. 1:**
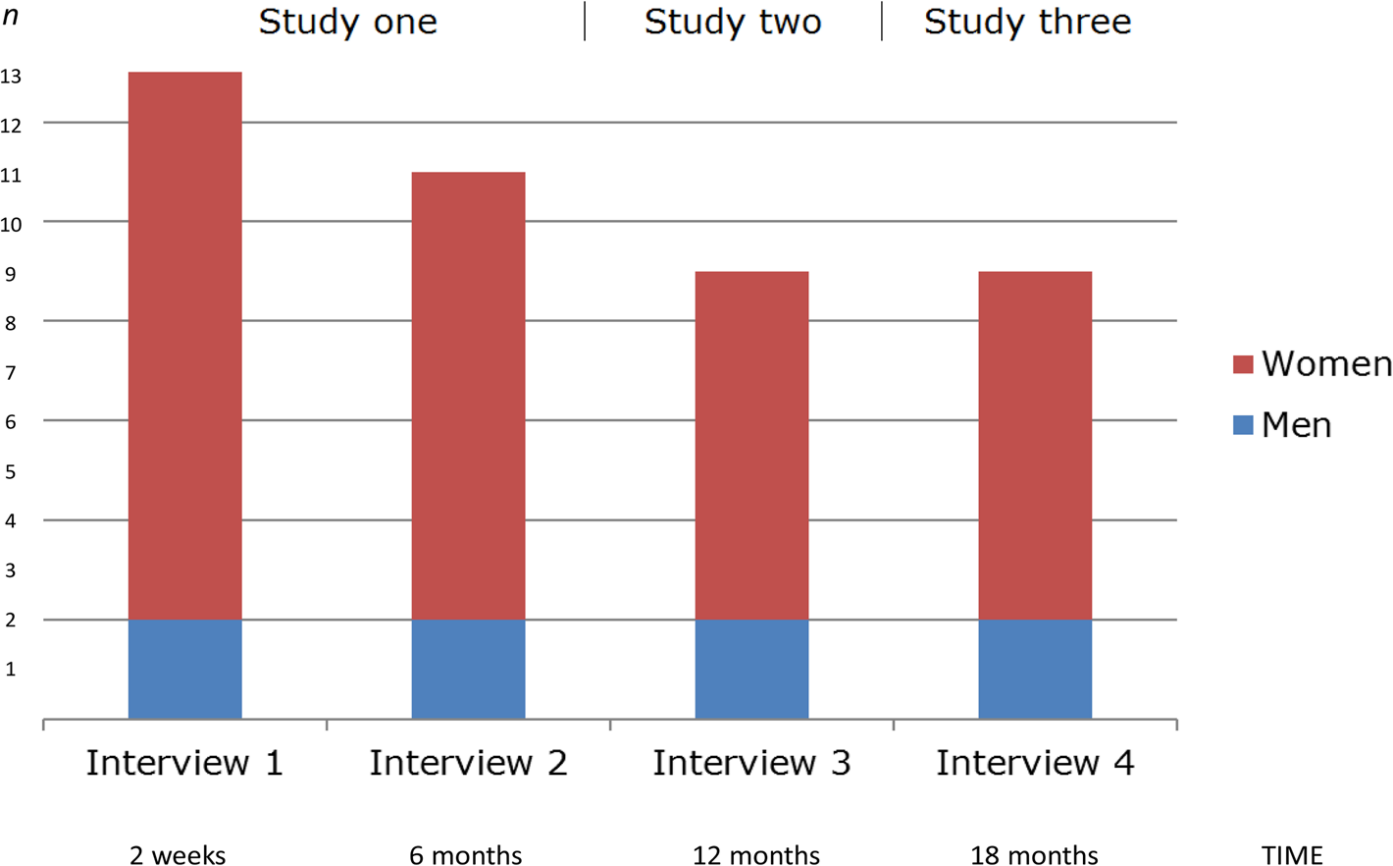
Longitudinal design and number of participants at four interviews

This study reports findings from individual interviews 18 months after a HF. It is the third and final part of a longitudinal qualitative study following the same group of aged adults through four interview rounds (Fig. 1). The development, change, and continuity during the 18 months were reported in a PhD thesis [32]. The first study [33] used data collected at 2 weeks and 6 months after HF to explore barriers and facilitators for being active. The second study explored experiences of being active after 1 year, a time where improvement of functional ability most likely is no longer possible. The repeated encounter with participants through serial, individual interviews allowed for more personal relationships and gave the researcher a profound understanding of their whole situation, adding depth to the analysis [34]. The study is registered in the Central Denmark Regional Research Council journal no. 1–16–02-422-15.

### Participants

Nine male and female survivors of initially 13 participants giving written informed consent while still in hospital due to a HF agreed to meet with the first author a fourth time (Fig. 1). Inclusion criteria were age ≥ 65 years with pre-fracture dependency on help and / or dependent on a walking aid prior to the HF [35]. Participants were aged between 72 and 94 years, and all except one experienced further decrease of physical functioning due to the HF. Three participants had died and one withdrew due to poor mental health.

### Data collection

Data were collected using semi-structured interviews. The first author met with participants in their own homes between January 2nd and March 8th 2018. The Apple® smartphone app “Memos “was used to record the interviews. Topics from former interviews needing more elaboration to be fully understood helped to inform the interview guide developed for this study only [see Additional file 1]. All participants were still living in the same place except Participant 4, who had suffered a stroke approximately 6 months earlier, and now heavily dependent on help, had moved from her flat into a nursing home. Her interview was the shortest, lasting for 32 min. The other interviews lasted between 48 and 79 min. The first author felt welcomed as a familiar person, e.g. being invited for lunch, or being allowed to enter even though the visit had been forgotten and the house was messy. Some wanted to fill the gap, up-dating the researcher on what had happened since the last interview, some asked about the interviewer’s personal life, and some showed things related to what they were talking about, e.g. pictures, presents, books. The semi-structured interview guide based on open-ended questions had questions focusing on looking back and questions on experiences of being active, i.e. situations that were difficult or situations with other people. The opening question “How are you doing “invited participants to talk about what was important in their life right now and was accompanied by double follow-up questions: questions used to secure an atmosphere of sharing an interest in the subject dealt with and questions to access knowledge [31]. Follow-up questions, silence, repeating words, and nodding were used to direct the attention toward a deeper meaning of experiences [36]. Questions referring to concepts (e.g. “Please tell me about your biggest limitation for being active“) left some participants silent without an answer, whereas re-phrasing the question into an invitation to tell about an experience (e.g. “Please tell me about a situation where it was difficult to be active“), they began to tell about something that had happened in their everyday lives.

### Analysis

The analysis was interpretive and used data from the present interview, whereas data from previous interviews were part of the researchers pre-understanding. Staying close to the phenomenon as revealed by participants, a hermeneutical circular movement between the parts and the whole was applied, which involved looking back on themes and selected transcripts from former interview rounds. To further a critical stance regarding the researchers’ pre-understandings, the interpretative process was prolonged. It was guided by curiosity and openness toward what the data revealed about experiences of being active, reading and re-reading transcripts and the literature [31]. Transcribed interviews were analyzed following five steps of meaning condensation as described by Kvale [36]. 1) All interviews were listened to, and transcripts were re-read to get a sense of the whole account of each participant. 2) Meaning units expressing a connected whole were selected and condensed into more essential statements. 3) Condensed meaning units were rewritten to obtain a more abstracted understanding. 4) Considering the research question, the rewritten meaning units were challenged and reconstructed. 5) Meaning units were interpreted and written into themes and sub-themes expressing diverse experiences. Interpretations questioned in discussions and reflections with co-authors leading to new insights supported credibility and dependability [30].

## Results

For most participants, the HF had been a serious event that changed their everyday lives and their ability to manage. Now, 18 months later, the changes in their level of activity due to the HF were a fundamental worry but were becoming part of their being (Table 1). For some, other diseases had changed their possibilities for being active and were now the primary concern. Looking back to how they were before the HF event did not make much sense for some participants. It could be difficult to distinguish what had happened when. Rather than answering direct questions about what their lives had been like 18 months ago before the HF, some participants drifted into stories about how their life was now, about their family, or they told stories about their life before the fracture. They preferred talking about their current situation, experiences of being with other people, and experiences of managing in everyday lives.

**Table 1:** Changes in being active due to HF or other diseases

Participant no.	Pre-fracture walking aid & need of help	Permanent changes due to the HF: - A fundamental worry (W) - Accepted as natural part of being active (A)	Other diseases - Overshadowing HF (O) - An additional worry (A)
1	Walker outdoor. Cleaning, medicine dosage	Less pain from the hip	Pain due to fracture of the humerus (O)
2	Walker Taking a shower, cleaning, laundry, medicine dosage	Pain from the hip (W) Dependent on using a walker all the time (W) Need help when taking a shower (A)	No
3	Walker Shopping	None	Increased tiredness due to progression of parkinsonism (O)
4	Walker outdoor	Pain from the hip (W) Unable to go walking for pleasure or go shopping up-town (W)	Wheelchair bound after stroke shortly after previous interview (O)
5	Walker outdoor away from the house Visits for security, cleaning	Unable to walk stairs and cannot visit neighbor friend (W) Unable to attend Bingo on her own (A) Unable to do own gardening (A)	No
6	Walker long distance outdoor Cleaning	Pain from the hip (W) Impaired balance and repeated falls (W) Unable to go dancing (W) Unable to drive a car (W)	No
7	Walker, once in a while without in the house Cleaning, laundry, medicine dosage	Slight pain from the hip (A) Permanent use of walker (A)	Increased dependency due to progressing loss of eye-sight (O)
8	Wheelchair, walker Transferring, personal hygiene, dressing (socks)	Wheelchair bound (A) Had to move into sheltered senior housing and live away from his wife (W)	No
9	Walker, support from wife Verbal guiding when transferring.	Pain, impaired balance, need help when showering (W)	Increased stiffness and reduced functioning of the leg due to episodes of transitory ischemic disease (A)

Two main themes (Table 2) describe participants’ experiences of being active in the context of everyday life. The main theme “Feeling the continuity of life “in four subthemes elaborates mainly on well-being experiences of meaningfulness, which are the experiences that are in the foreground when aged adults talked about their lives. However, experiences were intertwined with experiences of meaninglessness described in the main theme “Feeling vulnerable“.

**Table 2:** Two main-themes and six sub-themes

Main-themes	Sub-themes
Feeling the continuity of life	Gratitude for present possibilities Connected with earlier life-experience Thoughtfully managing vulnerability Belonging with other people
Feeling vulnerable	Thwarted Sad and regretting lost continuity in life

### Feeling the continuity of life

Embodying vulnerability and in the face of being toward death, participants were concerned about their possibilities for being active. Feeling the continuity of life was a sense of gratitude for present possibilities for being active, feeling supported by their own thoughtfulness and earlier life-experiences, and a sense of moving forward while feeling connected to other people.

#### Gratitude for present possibilities

Gratitude for present possibilities was a feeling paradoxically adding to a sense of moving forward and being connected to the temporal continuity of life. Living with uncertainty and the awareness that future possibilities for being active could be limited and vulnerability might increase, worrying about the future did no good. When talking about the future and the risk of further decrease of physical functions, Participant 7 said that “.*.. I’m not going to spend time wondering about that. […*] *that’s no use.* “Taking 1 day at a time, feeling gratitude for and appreciating the things they were still able to do was essential to feeling part of the continuity of life. These experiences ranged from Participant 8 appreciating the freedom possible after moving to a nursing home: “*I’ll tell you, going to the bathroom by myself, that is wonderful! And brushing my teeth! But the bathroom, that’s important, it’s so nice because I don’t have to call for someone,* “to Participant 6 exclaiming: “*Well, I’m happy every time I can solve a task.* “For Participant 1 after having been close to dying gratefulness was talked about as a feeling always present in the background. Participant 7, who was almost blind and could not go out on her own, seemed sincerely content, never talking about any imprisonment, or regretting her situation. For her, not being able to manage independently, a good life implicated gratitude toward “... *that I have good helpers coming here.* “Being able to stay in their homes or do things was not a matter of course but was appreciated as a gift of a prolonged possibility for feeling independent and free. Participant 5 was talking about going shopping in the supermarket and when asked what was so good about it she replied: “*then I know ‘you can do it yourself‘.* “In the act of doing, participants confirmed that it was still possible to do it and by focusing on what they could do, they maintained a silent hope for being able to continue being able to do it, also in the future.

#### Connected with earlier life-experiences

Feeling connected with earlier life experiences was related to a sense of meaningfulness in places, allowing a sense of feeling at home with oneself. This was revealed in words, but also by showing a special book, things, and photos in the home to the interviewer. Since childhood, Participant 6 had been going to the beach. Now, despite using a walker, she still enjoyed walking on the beach, priding herself on the agility necessary to maneuver on uneven ground: “*I hum, feel myself being able to do a little of what I was able to before. I just have to think about how to do it. I didn’t have to do that before, I’d just go, just walk..* “For Participant 9, an excursion with the day-care-center to his former workplace which now was turned into an Exploratorium was: “*...one of the biggest experiences I’ve ever had (cries)*.“.

Feeling connected with personal capacities developed through life, participants had a sense of dignity. By using and developing creativity, perseverance, and patience in difficult to manage everyday situations, participants were able to endure and keep on going. In waiting time for her son to come by and help her, Participant 3 was telling about the difficulties in trying to move a table and a carpet so she could wash the floor before he showed up. With a little laugh she said “*I got it done*! “Keeping on making an effort for most participants was part of a sense of dignity, living up to life-long values, as also expressed by Paul who had learned from his brother: “*You don’t give up.* “Some participants also came to accept dependency as natural and part of becoming old. They aligned with limitations and with the fact that keeping on trying was too exhausting and some things were impossible to accomplish. This was the case for Participant 5, who at a previous interview was exhausting herself by trying to manage her own garden: “*when you have to do it, you can* “(interview 3). Now she had hired a gardener. Talking about herself with a smile she explained “*she has gotten too old.* “When asked how she felt about that she was without regrets: “*Well, I am going to be 87 next time after all.* “Feeling connected with earlier life experiences and acknowledging one’s own stamina through life, it was possible to build on ones self-esteem and self-confidence.

#### Thoughtfully managing vulnerability

Thoughtfully managing vulnerability was an experience of well-being, maintaining a sense of identity and courage. It was a call from one’s conscience to do things, to care about one’s own being and stay independent. Relieving the strain in everyday life and increasing a sense of safety and self-confidence were essential, and the participants’ many experiences of how this was done are presented in Table 3. Paying attention to the present, the body and the surroundings, participants had learned how to move with care to avoid falling and pain and using unnecessarily amounts of energy. During the interview, Participant 5 went to her bedroom and brought back a device for putting on socks, stating that “*it’s helped me a lot.* “Being vulnerable, some risks were not worth taking and participants avoided walking on stairs when they were on their own. Each moment could call for thoughtfulness. It was a balance between being active and being careful, as stated by Participant 7: “*I also have to be careful not to push it […*]. *You have to use the strength you have for different things. So you take your walker and so on*. “It was a new way of moving, and in earlier interviews, Participant 3 and Participant 6 expressed that it had been difficult, but now they could avoid falling. Discovering possibilities for being active in a safe way could involve civil disobedience; not sure whether she was allowed to use the walker (a property of the government) when she went swimming in the ocean, Participant 6 concluded: “*It helps me swim just as much as it helps me walk!* “Participants felt responsible to stay healthy. Participant 6 also did exercises in bed every morning, for it was easier when the body was still warm and afterwards she was*:* “*... able to happily jump around throughout the day.* “For some participants, managing vulnerability implied maintained hope of progress and of again being able to resume or more easily manage everyday life. For Participant 2, hope of progress encouraged her zest for life: “If I didn’t have that (hope), I might as well … sit here and wait for it to end. “For Participant 2, Participant 3, Participant 6, and Participant 9 to be able to manage their vulnerability, they needed help, e.g. receiving massage, or attending physiotherapy, for effective training and knowledge about the body, thereby making it easier to move and increase their self-confidence in the ability to manage.

**Table 3:** Experiences of strain relief and increased self-confidence

Relieving the strain in everyday life	Increasing a sense of safety and self-confidence
Modifying the home environment with help from municipality staff	Being in familiar places
Using assistive devices	Receiving help
Using routines and procedures	Being near other people
Having easy access to things	Exercising in the home or in groups
Going for walks or going swimming	Training with physiotherapist • Effective and demanding training in a safe environment • Knowledge about moving safely
Receiving massage	Avoiding walking on stairs, in the dark and on icy surfaces

#### Belonging with other people

A feeling of belonging with other people was something to look forward to. Sharing experiences and talking with other people provided a sense of something more in life than managing everyday chores. When, in the middle of struggling to move a rug beneath her sofa, Participant 3 had an unexpected visit from her grandson and his girlfriend, she declined their offer to help, preferring to be updated on their lives and whereabouts: “*‘You are absolutely not helping me’, I said, ‘you are going to have your coffee’.* “Participants were drawn toward being with other people in mutual relationships. It could require a lot of energy to socialize, but it was worth the effort of getting ready, getting out of the house, and getting in and out of a car. Being active was more fun when they had a sense of belonging. For Participant 2, walking alone just for the sake of walking, “*it does absolutely nothing for me*, “whereas being together with other people, it was easier. The attention was on being with other people and their common experiences. The interaction, what was happening was in the foreground, paying less attention to the act of being active. For Participant 6, meeting new people was essential: “*And I walk a lot. And what brings me the most joy these days is (talking to other people) […*] *I’m so chatty, you know, and I say hello to the people I meet and if they start talking to me, I will talk to them.* “In contrast, other participants preferred being with people they knew, with whom they had a sense of belonging. Participant 8 lived in a nursing home, spending his weekdays in the adjacent daycare center; he enjoyed talking with people, helping out serving coffee but didn’t join in on the activities: “*... They go on outings, but that*’*s ok, I enjoy going with my son just as much; we talk about all sorts of things*. “People who had known them for a long time provided a sense of safety and were a natural refuge from chaos, offering help with practicalities as well as sharing life experiences of successes and failures. Family and friends yielded possibilities in life; going on excursions seeing new places, inviting for birthdays, helping to organize and carry out a birthday party for friends. Although participants were grateful to receive help, they also were leaning on the relationship as a natural thing, part of the lifelong mutual commitment of a family or close friendships. As expressed by Anna, whose daughter helped out with shopping, “*well, that’s how it’s supposed to be*.“.

Being together with healthcare providers, a feeling of belonging was an experience of feeling respected, a sense of community, and feeling safe and taken care of. When the help or advice they received was related to problems they were experiencing in their everyday lives, they felt acknowledged as dignified human beings.

### Feeling vulnerable

Feeling vulnerable was mainly experienced in association with the experiences elaborated in the previous main-theme (“Feeling the continuity of life“), as experiences of *avoiding* feeling pulled toward greater vulnerability. In this main theme, feeling vulnerable was rooted in more explicit experiences. Two subthemes “feeling thwarted “and “lost continuity in life “detailed how managing everyday life was an ongoing struggle with uncertainty and unpredictability, and participants seemed to endure these experiences on their own.

#### Thwarted

Feeling thwarted when receiving help because of healthcare providers’ lack of human perspective or knowledge was an experience of loss of dignity/meaninglessness, e.g. when meeting with specialists, who focused only on symptoms and were not interested in how they were doing and the possibilities they had for being active; or when homecare ignored their limitations and needs. Participant 7 had one helper who continuously told her to do the dishwashing after lunch; however, being almost blind she felt helpless, unable to see whether the tableware was clean. Participant 6 felt thwarted by helpers, for example, when they left a mess or did not respect the way she was trying to organize things to make them easier to manage. Participant 6 had succeeded in maneuvering boxes of diapers into the bathroom, stacking them so they looked nice and were easy accessible from the toilet. Next morning a helper tore open one of the boxes, letting the diapers spill out, and another helper *“...had been so clever as to put them […*] *on the shelf behind my toilet […*] *which means I have to go all the way behind and then up and reach for one of the diapers. You know what, I can’t do that!* “She had given up telling them how she wanted things done because she knew *“from experience how insulted they can get and then you’ll get a snappy response.* “For some, feeling thwarted was an experience of needing training, but having difficulties attending training sessions due to distance or weather difficulties. Feeling thwarted was also an experience of lacking knowledge about assistive devices. For Anna, this meant lying in the floor a whole night after a fall, not sure whether helpers would hear her emergency call during night, and Participant 6 although longing to go to a market in a neighboring village, only used her electric scooter for short trips because she did not know the capacity of the battery.

#### Sad and regretting lost continuity in life

When possibilities for being active were reduced, participants felt sad, regretting lost continuity with who they used to be. Feeling exhausted and embodying weakness, pain, and instability, a sense of unpredictability could prevent participants from doing what they wanted and struggled to do. A part of who they were was what they were able to do and accomplish, and when they could not do something, they felt that an identity as an independent, active, and persevering person was lost.

The two men talked about lost possibilities for outdoor activities; the women mostly about household chores; both men *and* women talked about limited freedom and limited possibilities for socializing. For Participant 6, to be able to look at things happening in her surroundings while sitting on her terrace in her bath robe and drinking a cup of tea, she had to remove dead plants: “*I do all I can, I have been removing withered plants but then it comes to a halt; I can pull them up and gather them but I can’t carry them away ... it annoys me*. “She would feel exhausted and when sitting down for a cup of tea, she would fall asleep; even after a rest, she would not have the stamina to carry on. Unable to drive her car, she was unable to go shopping outside her local community, and she also depended on public services to attend social activities. Not accepting her own limits, Participant 6 was full of regrets: “*Then I think to myself ‘well, you are an old weakling when you can’t even do that*. “Participant 2 continuously regretted her lost possibilities for helping out in the house, and feeling impatient to see results from her training she said: “*I think I should be getting more mobile faster ...It can’t be right that I won’t be able to do some things, even though I’ve gotten old.* “Life had changed, and it was difficult to accept. In contrast, seeing no alternative and also handicapped due to other diseases, Participant 3, Participant 7, and Participant 8 had resigned and come to accept changed life conditions and things no longer possible to do. However, they talked about their loss with sadness, e.g. when Participant 8 was telling that: “*There are many things, I want to do […*] *I arranged roses, made flies for the fishermen […*] *I was out catching trout and herring and plaice; there was something going on every minute, but that’s over now*“. Participant 3 choked up when stating that “*I’m used to being able to do everything; that’s probably what’s difficult to accept*. “She reluctantly accepted a need for increased help from her family: “*I’m going to have to accept that when I’m not able to do some things myself.* “Although almost blind and not able to manage her house or go out, Participant 7 never complained; “*that’s how it is* “and “*that’s how it goes* “was a common reply from her, for example, when talking about disabled friends who were not able to visit, or friends who had passed away. However, her occasionally quiet voice or how she looked down at her hands was interpreted as sadness. For one participant, a sense of continuity in life had stopped. Participant 4 had lost a daughter to cancer, had suffered a stroke, and now, wheelchair-bound, had moved into a nursing-home. “*I have nothing more to tell* “was one of her last remarks. Being together with other residents with whom she felt no kinship did not bring any joy, and unable to get into a car, she missed going on her usual trips with a friend.

## Discussion

This study explored the meaningfulness of being active from the perspective of aged adults with pre-fracture dependency 18 months after HF. The findings in this study confirmed that they embodied vulnerability and that their possibilities for managing everyday life tasks and for participating in social activities were reduced [3]. In this situation, challenging the meaningfulness of being active, well-being was a vital resource.

Focusing on daily life experiences, this study adds to practices inspired by the concept of reablement [28]. Some healthcare providers aiming to close the gap between recommended and actual activity levels in aged adults 18 months after HF may primarily be focusing on the progress of functional ability and independency. This can be meaningful for some, which in this study was the case for participants for whom meaningfulness was connected with well-being as a temporal experience, a hopeful orientation toward progressing future possibilities for better physical functioning. Without this hope, a felt invitation to be active or the motivation to do so was missing. The findings are in line with earlier studies describing that a hope for progress increased motivation to be active and to exercise [37], and when hope and belief in the future were missing, being active was meaningless [13]. In our study, feeling hope was a resource that helped participants keeping on struggling and trying. However, in contrast to Gorman’s study (2013), to be continuously focusing on progress and keeping on struggling was for some was an experience of suffering. This finding seems to be in alignment with what Galvin & Todres (2011) described as an experience of an “elusive present”, a feeling of being unable to appreciate the present possibilities for being active. This also seemed to apply to aged adults 18 months after HF.

This study, being part of a longitudinal study, expanded on the understanding of how reappraisal of meaningfulness was an experience of developing possibilities for well-being through time. During the first 6 months after HF, hoping for progress was essential, and when progress was slow or absent, it could bring suffering [33]. Around 1 year after HF, hope for progress was still an issue. However, exploring how it was possible to be active in the proximity of death, the future could be anxiety provoking or not possible to grasp. Participants were in the process of moving toward settlement and reconciliation with their loss and limitations. In the present study concerning experiences 18 months after HF, hope for progress was still essential in order to experience meaningfulness. However, for most participants, hope also involved experiences of being at peace, a sense of gratitude, and appreciation of present possibilities. Being thoughtful and managing vulnerability was part of the process. In an earlier study on the fear of falling, the significance of being thoughtful entailed carefulness, a protective strategy making it possible to be active and maintain an identity as an independent person, rather than a person at risk of falling. Aged adults adapted and found a balance between feeling safe and remaining active [38]. Acceptance can be an ongoing process restoring a sense of normality [14]. Our study further elaborated on how aged adults in trying over and over again were involved in a process of understanding. In the act of doing things during 18 months after HF, aged adults were discovering possibilities for being active while finding resources in the experiences of dwelling. It was a process of being thoughtful, caring about one’s own safety, resources, and health, and thereby becoming aligned with their changing life situation. This did not mean that suffering was eradicated, but it was endured and came to be accepted, and “being at one with what is there” [1] was possible over time. Being active with a sense of gratitude toward present possibilities, the aged adults in this study maintained a sense of meaningfulness. This way, the experience of life coming to an end did not prevent them from making sense of being active. This finding added to the concept “temporal dwelling” in the theoretical framework of well-being [25]. Opposed to the suffering experience of a blocked future, gratitude was a way of feeling connected with their own being, feeling “brought home” to the very simple event of “just being” [25]. On the other hand, when aged adults in this study were unable to align with limitations and loss, it could result in suffering from being without “dwelling” [39]. This was illustrated in this study as an experience of hopelessness related to a blocked future: feelings sad and regretting loss; feeling unable; and feeling exhausted, incapable of physically handling daily tasks.

Searching for and supporting possibilities for the well-being of inter-subjective experiences that are valuable for the individual and “an invitation into a welcoming future” [25], healthcare providers may convey to aged adults the energy to be active. For all participants in our study, hope and future possibilities mainly were related to inter-subjective experiences: looking forward to experiences together with other people. Similar findings were reported in a systematic review on aged adults’ experiences of being active [40] and in studies on aged adults’ experiences 1 year after a HF [13, 19, 35]. Social interaction can be a meaningful way for aged adults to challenge the limits of their ability [38], and aged adults may benefit from having fun and to be in a trust-based atmosphere [33]. Our study added that to be active, it seems important for aged adults to have something to look forward to. Even small changes mediated through relationships with other people can contribute to this well-being experience. The importance of feeling kinship, as well as the vitalizing experience of looking forward to experiences with other people have been recurring themes during the four interview rounds in which the same participants were interviewed during 18 months after HF. In all phases after HF, other people were essential for aged adults’ possibilities for being active, as practical support and help, and as a source of feeling connected with a meaningful future, enduring the awareness of being mortal.

Person-oriented care is needed for aged adults to feel supported when the meaningfulness of being active is challenged in various ways [26]. Aged adults in this study seemed to endure the ongoing uncertainty and unpredictability when being active in everyday-life situations on their own. Furthermore, the aged adults who were dependent on help from healthcare providers had experiences of feeling thwarted when staff did not recognize the problems they were struggling to solve or who they were as people. On the other hand, receiving help to make sense of being active, feeling safe and respected supported a sense of meaningfulness. Epistemological reflections together with the ontological perspective of Uhrenfeldt et al. (2018) were argued to be fundamental when building a strong relationship where people in need trust and follow healthcare providers’ advice [26]. This study’s findings pointing to well-being as a resource can be helpful for healthcare providers building consensus in their development of guidelines for municipality-based rehabilitation, e.g. planning activities in a day-care center or developing activity focused home-visits [29]. Healthcare providers who focus mainly on being effective and regard the aged adults as consumer, risk adding to the suffering of aged adults [41], whereas emphasizing well-being can touch upon a motivation that appeals to the hearts of aged adults’ and that therefore may be more evocative than rational knowledge [42] and may represent a relief, making it easier to be active in the presence of suffering.

### Strength and limitations

This study aimed to explore aged adults’ experiences of being active 18 months after HF. The phenomenological-hermeneutic approach was found a strength, foregrounding the existential meaning of their experiences. Using the philosophy of Heidegger and the theoretical framework of well-being provided a deeper understanding of how not only functional impairment but also existential concerns regarding mood, relationships, identity, and places seemed to play a central role for aged adults striving to be active after HF.

The authors know of no other studies with this specific focus or ones in which interviews were carried out at this time-point after HF. It may seem a limitation that our discussion regarding the relevant literature to a large extent concerned studies on experiences of being old and living with disability and chronic illness. However, this emphasizes that 18 months after HF, aged adults do have challenges similar to those of other people. Based on the rich descriptions of participants’ experiences in this study, transferability to aged adults without HF is possible. It should be taken into consideration that the study took place in a country where healthcare and older-care services are largely tax-financed; that all women in the study were widowed, while the two men were married; and that no participants were living in large cities. Aged adults living in other cultural settings with different social conditions and living in other geographical settings may have different experiences. It could be a limitation that all participants in the study had other diseases that could have affected their answers to the interviewer’s questions. However, in human life the meaningfulness of being active is a unified experience. Different life-conditions related to the person, to other people, and to the environment, already influence the experiences people have [21]. Having met with participants three times previously made it possible to consider the significance of other diseases as part of the challenges aged adults were experiencing 18 months after HF.

It was a strength that the study was part of a longitudinal study. First, a trustful relationship was developed over time, which, particularly in vulnerable aged adults, is considered vital to avoid omission of important messages [43]. Second, two former studies based on the experiences of the same participants allowed the researchers to compare the present study results with two former studies, which helped provoke and transcend own pre-understandings [30]. The pre-understandings of the first author, a physiotherapist with three decades in-hospital practice experience, were both a limitations and a strength, and questions about functioning and the use of assistive devices did occasionally come up. However, having prepared an interview guide, this pre-understanding was kept from dominating the interview. Rather, it did seem helpful in following up on what participants were talking about.

To bring forward new issues in the dynamics of a group, focus group interviews were considered an alternative to individual interviews [44]. However, considering the vulnerability of participants and their health concerns, i.e. tiredness, difficulties speaking, impaired hearing, and decreased eyesight, we found individual interviews to be appropriate.

Only nine participants were included in the study, which could be a limitation. However, within phenomenological-hermeneutic research traditions, small sample size is acknowledged to be suitable to secure in-depth knowledge [45] and avoid shallow interpretation [36]. Furthermore, knowing participants through 18 months changed and enlarged the horizon of the researchers, thereby allowing for a deeper understanding to emerge. Interviewing participants in their own homes added trustworthiness to the analysis, providing a possibility to see how they moved about in their familiar surroundings. This also gave participants the opportunity to show personal items and share stories related to them. This added to a deeper understanding of the meaning of being active in daily life situations [2].

## Conclusion

This study offers in-depths insights into aged adults´ experiences of the meaningfulness of being active 18 months after HF. The findings emphasize that changes in their mobility still exist and that it is a struggle to be active in meaningful ways. Aged adults have to endure being vulnerable and are aware of the nearness of death. Maintaining hope is essential, and it is vital to restore a sense of continuity with who they used to be. After 18 months, aged adults with a HF may still be in need of help to make everyday life easier and to build on a sense of safety and self-confidence. Additional training can be supportive; however, without appreciation of the present possibilities, keeping on struggling for progress can bring more suffering. Healthcare-providers must pay special attention to well-being experiences in terms of feeling at peace, feeling gratitude and kinship with other people, and restoring a sense of normality. Such experiences can be an essential resource and bring energy when aged adults struggle to find meaning in being active. The findings can guide development and testing of interventions to support aged adults in being active after HF.

## Supplementary information

**Additional file 1.** Interview guide. Interview guide developed for this study.

## Data Availability

The data supporting the conclusion of this article may be available from the corresponding author upon reasonable request.

## Abbreviation

HF: Hip fracture
