# Supplementary material for: Being active 1½ years after hip fracture: a qualitative interview study of aged adults’ experiences of meaningfulness
Source: BMC Geriatr. 2020 Jul 29;20:263. doi: 10.1186/s12877-020-01666-w (PMC7391487; doi:10.1186/s12877-020-01666-w)
Supplement: Supplementary file 1 — Additional file 1. Interview guide. Interview guide developed for this study. [file 12877_2020_1666_MOESM1_ESM.docx]

| Interview guide 18 months after HF |
| --- |
| How are you?  Looking back on the last 1½ years;   - - Can you please tell me about some good experiences?   - Can you please tell me about some bad experiences?   - What has changed in your life from before you had a HF?   - How do you feel about these changes?   Can you please give some examples of when you are physically active?  How do you feel about being physically active? What is most important for you to be able to do?  What possibilities do you have for being physically active? Do you experience limitations?  In which situations do you find it especially difficult to be active?  Can you please tell me about a positive experience where you were active and where you were together with other people? |
